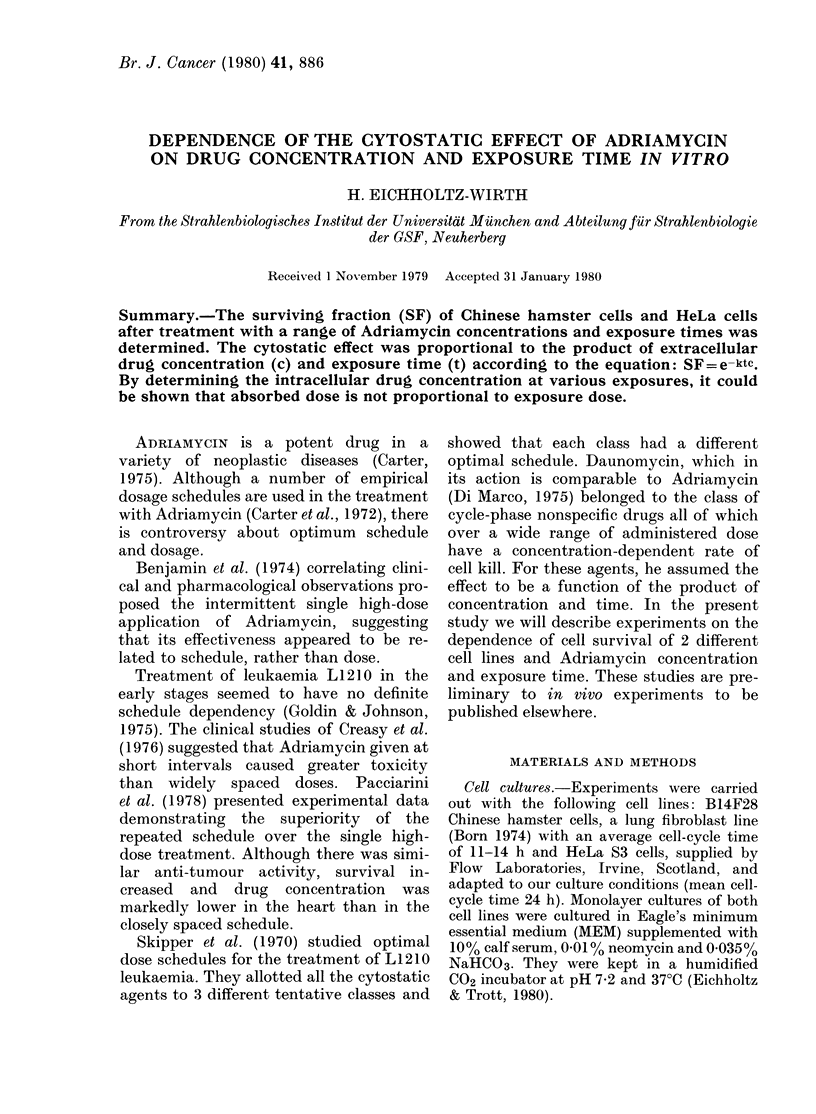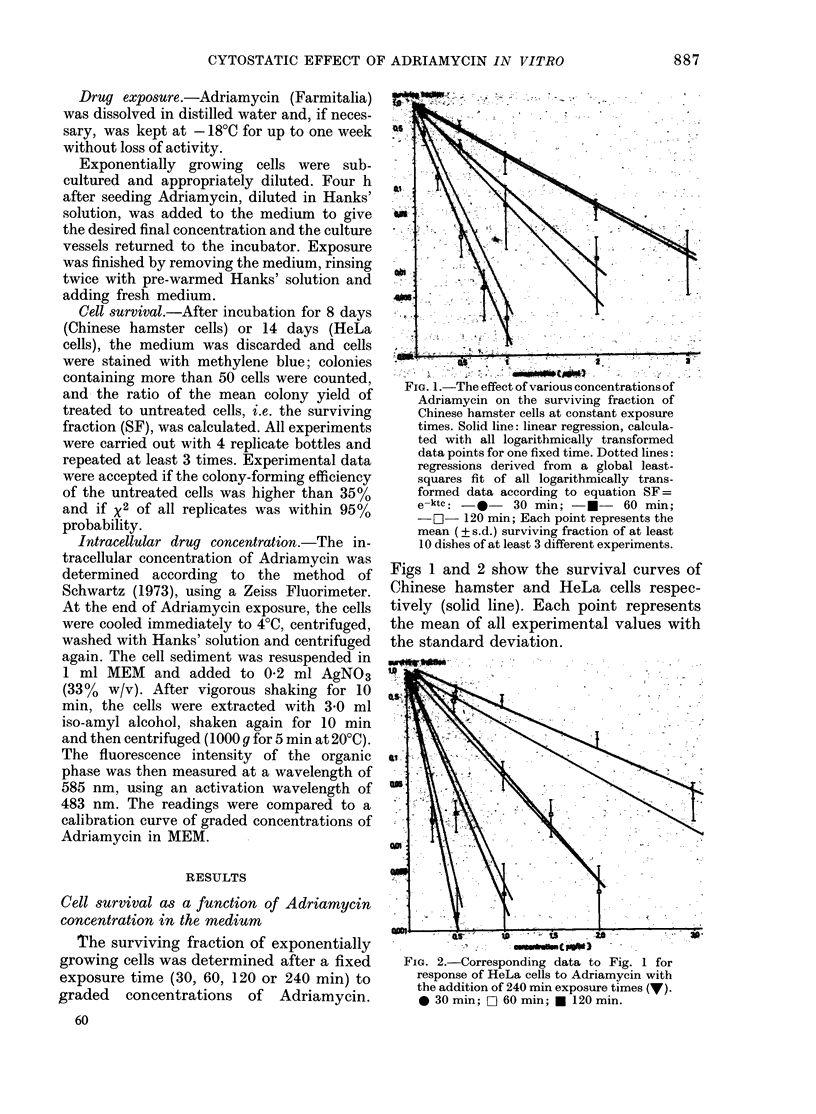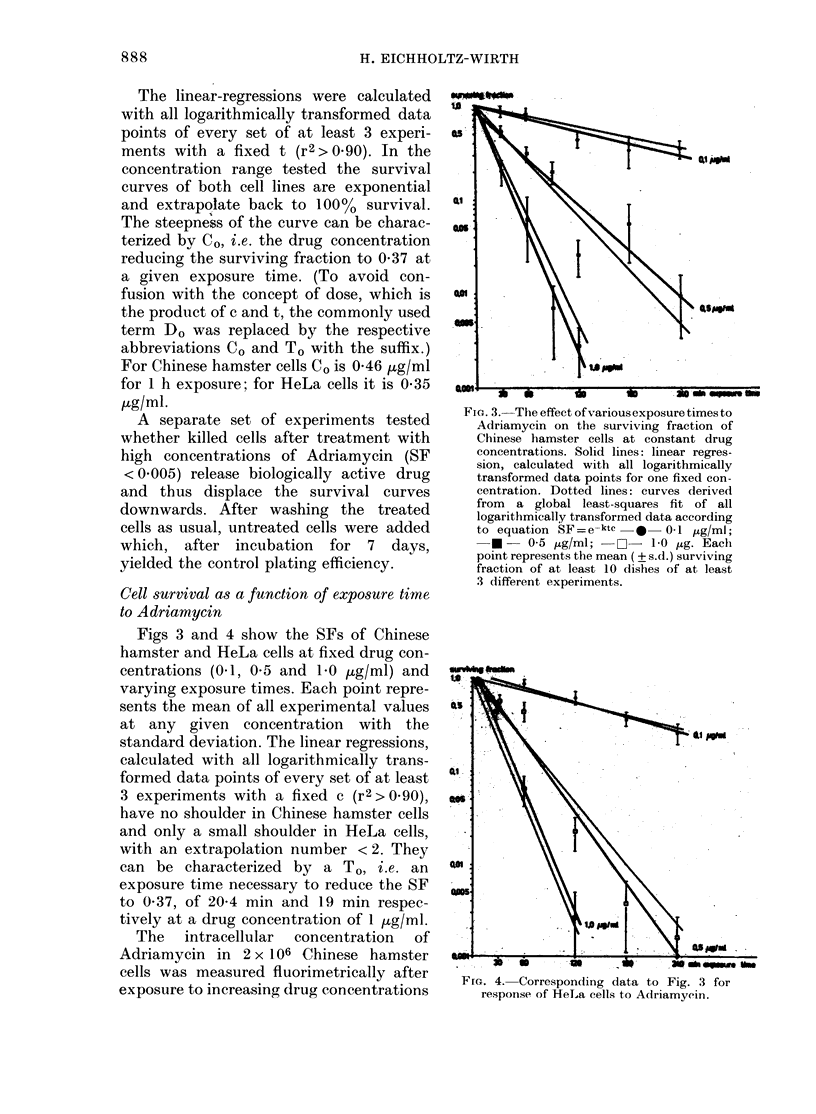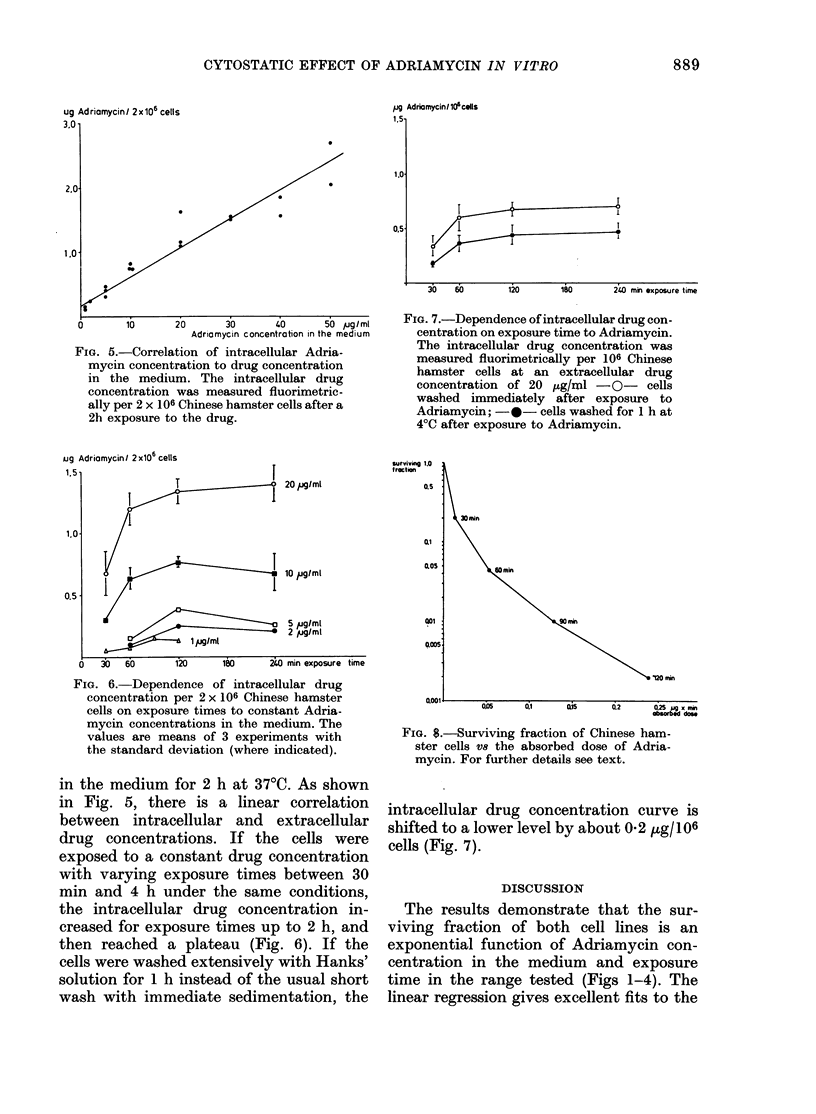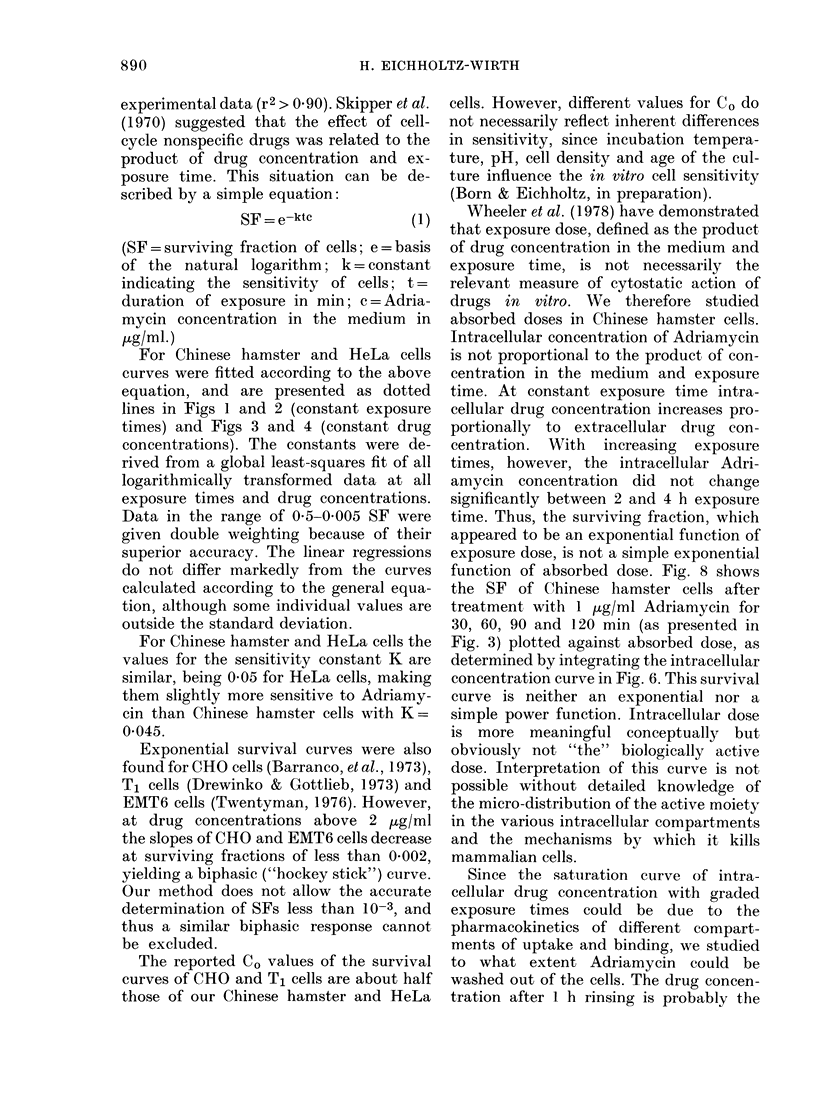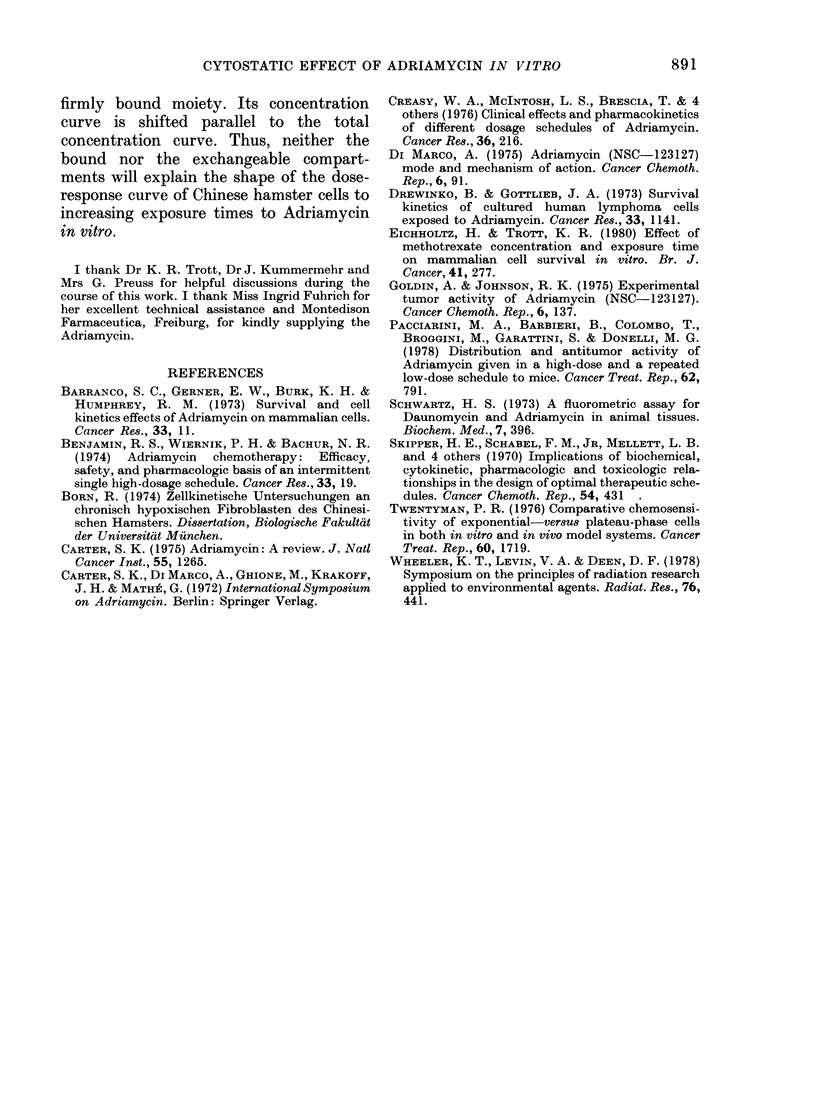# Dependence of the cytostatic effect of adriamycin on drug concenration and exposure time in vitro.

**DOI:** 10.1038/bjc.1980.165

**Published:** 1980-06

**Authors:** H. Eichholtz-Wirth

## Abstract

The surviving fraction (SF) of Chinese hamster cells and HeLa cells after treatment with a range of Adriamycin concentrations and exposure times was determined. The cytostatic effect was proportional to the product of extracellular drug concentration (c) and exposure time (t) according to the equation: SF=e-ktc. By determining the intracellular drug concentration at various exposures, it could be shown that absorbed dose is not proportional to exposure dose.


*Br. J. Cancer* (1980) **41**, 886

### DEPENDENCE OF THE CYTOSTATIC EFFECT OF ADRIAMYCIN ON DRUG CONCENTRATION AND EXPOSURE TIME *IN VITRO*

H. EICHHOLTZ-WIRTH

*From the Strahlenbiologisches Institut der Universität München and Abteilung für Strahlenbiologie der GSF, Neuherberg*

Received 1 November 1979 Accepted 31 January 1980

**Summary.—The surviving fraction (SF) of Chinese hamster cells and HeLa cells after treatment with a range of Adriamycin concentrations and exposure times was determined. The cytostatic effect was proportional to the product of extracellular drug concentration (c) and exposure time (t) according to the equation: $SF = e^{-ktc}$. By determining the intracellular drug concentration at various exposures, it could be shown that absorbed dose is not proportional to exposure dose.**

ADRIAMYCIN is a potent drug in a variety of neoplastic diseases (Carter, 1975). Although a number of empirical dosage schedules are used in the treatment with Adriamycin (Carter *et al.*, 1972), there is controversy about optimum schedule and dosage.

Benjamin *et al.* (1974) correlating clinical and pharmacological observations proposed the intermittent single high-dose application of Adriamycin, suggesting that its effectiveness appeared to be related to schedule, rather than dose.

Treatment of leukaemia L1210 in the early stages seemed to have no definite schedule dependency (Goldin & Johnson, 1975). The clinical studies of Creasy *et al.* (1976) suggested that Adriamycin given at short intervals caused greater toxicity than widely spaced doses. Pacciarini *et al.* (1978) presented experimental data demonstrating the superiority of the repeated schedule over the single high-dose treatment. Although there was similar anti-tumour activity, survival increased and drug concentration was markedly lower in the heart than in the closely spaced schedule.

Skipper *et al.* (1970) studied optimal dose schedules for the treatment of L1210 leukaemia. They allotted all the cytostatic agents to 3 different tentative classes and showed that each class had a different optimal schedule. Daunomycin, which in its action is comparable to Adriamycin (Di Marco, 1975) belonged to the class of cycle-phase nonspecific drugs all of which over a wide range of administered dose have a concentration-dependent rate of cell kill. For these agents, he assumed the effect to be a function of the product of concentration and time. In the present study we will describe experiments on the dependence of cell survival of 2 different cell lines and Adriamycin concentration and exposure time. These studies are preliminary to *in vivo* experiments to be published elsewhere.

### MATERIALS AND METHODS

*Cell cultures.*—Experiments were carried out with the following cell lines: B14F28 Chinese hamster cells, a lung fibroblast line (Born 1974) with an average cell-cycle time of 11–14 h and HeLa S3 cells, supplied by Flow Laboratories, Irvine, Scotland, and adapted to our culture conditions (mean cell-cycle time 24 h). Monolayer cultures of both cell lines were cultured in Eagle's minimum essential medium (MEM) supplemented with 10% calf serum, 0·01% neomycin and 0·035% $NaHCO_3$. They were kept in a humidified $CO_2$ incubator at pH 7·2 and 37°C (Eichholtz & Trott, 1980).

*Drug exposure.*—Adriamycin (Farmitalia) was dissolved in distilled water and, if necessary, was kept at −18°C for up to one week without loss of activity.

Exponentially growing cells were subcultured and appropriately diluted. Four h after seeding Adriamycin, diluted in Hanks' solution, was added to the medium to give the desired final concentration and the culture vessels returned to the incubator. Exposure was finished by removing the medium, rinsing twice with pre-warmed Hanks' solution and adding fresh medium.

*Cell survival.*—After incubation for 8 days (Chinese hamster cells) or 14 days (HeLa cells), the medium was discarded and cells were stained with methylene blue; colonies containing more than 50 cells were counted, and the ratio of the mean colony yield of treated to untreated cells, *i.e.* the surviving fraction (SF), was calculated. All experiments were carried out with 4 replicate bottles and repeated at least 3 times. Experimental data were accepted if the colony-forming efficiency of the untreated cells was higher than 35% and if $\chi^2$ of all replicates was within 95% probability.

*Intracellular drug concentration.*—The intracellular concentration of Adriamycin was determined according to the method of Schwartz (1973), using a Zeiss Fluorimeter. At the end of Adriamycin exposure, the cells were cooled immediately to 4°C, centrifuged, washed with Hanks' solution and centrifuged again. The cell sediment was resuspended in 1 ml MEM and added to 0·2 ml $AgNO_3$ (33% w/v). After vigorous shaking for 10 min, the cells were extracted with 3·0 ml iso-amyl alcohol, shaken again for 10 min and then centrifuged (1000 *g* for 5 min at 20°C). The fluorescence intensity of the organic phase was then measured at a wavelength of 585 nm, using an activation wavelength of 483 nm. The readings were compared to a calibration curve of graded concentrations of Adriamycin in MEM.

### RESULTS

### *Cell survival as a function of Adriamycin concentration in the medium*

The surviving fraction of exponentially growing cells was determined after a fixed exposure time (30, 60, 120 or 240 min) to graded concentrations of Adriamycin.

Fig. 1.—The effect of various concentrations of Adriamycin on the surviving fraction of Chinese hamster cells at constant exposure times. Solid line: linear regression, calculated with all logarithmically transformed data points for one fixed time. Dotted lines: regressions derived from a global least-squares fit of all logarithmically transformed data according to equation $SF = e^{-ktc}$: —●— 30 min; —■— 60 min; —□— 120 min; Each point represents the mean (±s.d.) surviving fraction of at least 10 dishes of at least 3 different experiments.

Figs 1 and 2 show the survival curves of Chinese hamster and HeLa cells respectively (solid line). Each point represents the mean of all experimental values with the standard deviation.

Fig. 2.—Corresponding data to Fig. 1 for response of HeLa cells to Adriamycin with the addition of 240 min exposure times (▼). ● 30 min; □ 60 min; ■ 120 min.

The linear-regressions were calculated with all logarithmically transformed data points of every set of at least 3 experiments with a fixed t ($r^2 > 0{\cdot}90$). In the concentration range tested the survival curves of both cell lines are exponential and extrapolate back to 100% survival. The steepness of the curve can be characterized by $C_0$, *i.e.* the drug concentration reducing the surviving fraction to 0·37 at a given exposure time. (To avoid confusion with the concept of dose, which is the product of c and t, the commonly used term $D_0$ was replaced by the respective abbreviations $C_0$ and $T_0$ with the suffix.) For Chinese hamster cells $C_0$ is 0·46 μg/ml for 1 h exposure; for HeLa cells it is 0·35 μg/ml.

A separate set of experiments tested whether killed cells after treatment with high concentrations of Adriamycin (SF < 0·005) release biologically active drug and thus displace the survival curves downwards. After washing the treated cells as usual, untreated cells were added which, after incubation for 7 days, yielded the control plating efficiency.

FIG. 3.—The effect of various exposure times to Adriamycin on the surviving fraction of Chinese hamster cells at constant drug concentrations. Solid lines: linear regression, calculated with all logarithmically transformed data points for one fixed concentration. Dotted lines: curves derived from a global least-squares fit of all logarithmically transformed data according to equation $SF = e^{-ktc}$ —●— 0·1 μg/ml; —■— 0·5 μg/ml; —□— 1·0 μg. Each point represents the mean (± s.d.) surviving fraction of at least 10 dishes of at least 3 different experiments.

*Cell survival as a function of exposure time to Adriamycin*

Figs 3 and 4 show the SFs of Chinese hamster and HeLa cells at fixed drug concentrations (0·1, 0·5 and 1·0 μg/ml) and varying exposure times. Each point represents the mean of all experimental values at any given concentration with the standard deviation. The linear regressions, calculated with all logarithmically transformed data points of every set of at least 3 experiments with a fixed c ($r^2 > 0{\cdot}90$), have no shoulder in Chinese hamster cells and only a small shoulder in HeLa cells, with an extrapolation number < 2. They can be characterized by a $T_0$, *i.e.* an exposure time necessary to reduce the SF to 0·37, of 20·4 min and 19 min respectively at a drug concentration of 1 μg/ml.

The intracellular concentration of Adriamycin in $2 \times 10^6$ Chinese hamster cells was measured fluorimetrically after exposure to increasing drug concentrations

FIG. 4.—Corresponding data to Fig. 3 for response of HeLa cells to Adriamycin.

FIG. 5.—Correlation of intracellular Adriamycin concentration to drug concentration in the medium. The intracellular drug concentration was measured fluorimetrically per $2 \times 10^6$ Chinese hamster cells after a 2h exposure to the drug.

FIG. 6.—Dependence of intracellular drug concentration per $2 \times 10^6$ Chinese hamster cells on exposure times to constant Adriamycin concentrations in the medium. The values are means of 3 experiments with the standard deviation (where indicated).

in the medium for 2 h at 37°C. As shown in Fig. 5, there is a linear correlation between intracellular and extracellular drug concentrations. If the cells were exposed to a constant drug concentration with varying exposure times between 30 min and 4 h under the same conditions, the intracellular drug concentration increased for exposure times up to 2 h, and then reached a plateau (Fig. 6). If the cells were washed extensively with Hanks' solution for 1 h instead of the usual short wash with immediate sedimentation, the

FIG. 7.—Dependence of intracellular drug concentration on exposure time to Adriamycin. The intracellular drug concentration was measured fluorimetrically per $10^6$ Chinese hamster cells at an extracellular drug concentration of 20 µg/ml —○— cells washed immediately after exposure to Adriamycin; —●— cells washed for 1 h at 4°C after exposure to Adriamycin.

FIG. 8.—Surviving fraction of Chinese hamster cells *vs* the absorbed dose of Adriamycin. For further details see text.

intracellular drug concentration curve is shifted to a lower level by about 0·2 µg/$10^6$ cells (Fig. 7).

DISCUSSION

The results demonstrate that the surviving fraction of both cell lines is an exponential function of Adriamycin concentration in the medium and exposure time in the range tested (Figs 1–4). The linear regression gives excellent fits to the experimental data ($r^2 > 0{\cdot}90$). Skipper *et al.* (1970) suggested that the effect of cell-cycle nonspecific drugs was related to the product of drug concentration and exposure time. This situation can be described by a simple equation:

$$SF = e^{-ktc} \quad (1)$$

(SF = surviving fraction of cells; e = basis of the natural logarithm; k = constant indicating the sensitivity of cells; t = duration of exposure in min; c = Adriamycin concentration in the medium in $\mu$g/ml.)

For Chinese hamster and HeLa cells curves were fitted according to the above equation, and are presented as dotted lines in Figs 1 and 2 (constant exposure times) and Figs 3 and 4 (constant drug concentrations). The constants were derived from a global least-squares fit of all logarithmically transformed data at all exposure times and drug concentrations. Data in the range of 0·5–0·005 SF were given double weighting because of their superior accuracy. The linear regressions do not differ markedly from the curves calculated according to the general equation, although some individual values are outside the standard deviation.

For Chinese hamster and HeLa cells the values for the sensitivity constant K are similar, being 0·05 for HeLa cells, making them slightly more sensitive to Adriamycin than Chinese hamster cells with K = 0·045.

Exponential survival curves were also found for CHO cells (Barranco, *et al.*, 1973), $T_1$ cells (Drewinko & Gottlieb, 1973) and EMT6 cells (Twentyman, 1976). However, at drug concentrations above 2 $\mu$g/ml the slopes of CHO and EMT6 cells decrease at surviving fractions of less than 0·002, yielding a biphasic ("hockey stick") curve. Our method does not allow the accurate determination of SFs less than $10^{-3}$, and thus a similar biphasic response cannot be excluded.

The reported $C_0$ values of the survival curves of CHO and $T_1$ cells are about half those of our Chinese hamster and HeLa cells. However, different values for $C_0$ do not necessarily reflect inherent differences in sensitivity, since incubation temperature, pH, cell density and age of the culture influence the *in vitro* cell sensitivity (Born & Eichholtz, in preparation).

Wheeler *et al.* (1978) have demonstrated that exposure dose, defined as the product of drug concentration in the medium and exposure time, is not necessarily the relevant measure of cytostatic action of drugs *in vitro*. We therefore studied absorbed doses in Chinese hamster cells. Intracellular concentration of Adriamycin is not proportional to the product of concentration in the medium and exposure time. At constant exposure time intracellular drug concentration increases proportionally to extracellular drug concentration. With increasing exposure times, however, the intracellular Adriamycin concentration did not change significantly between 2 and 4 h exposure time. Thus, the surviving fraction, which appeared to be an exponential function of exposure dose, is not a simple exponential function of absorbed dose. Fig. 8 shows the SF of Chinese hamster cells after treatment with 1 $\mu$g/ml Adriamycin for 30, 60, 90 and 120 min (as presented in Fig. 3) plotted against absorbed dose, as determined by integrating the intracellular concentration curve in Fig. 6. This survival curve is neither an exponential nor a simple power function. Intracellular dose is more meaningful conceptually but obviously not "the" biologically active dose. Interpretation of this curve is not possible without detailed knowledge of the micro-distribution of the active moiety in the various intracellular compartments and the mechanisms by which it kills mammalian cells.

Since the saturation curve of intracellular drug concentration with graded exposure times could be due to the pharmacokinetics of different compartments of uptake and binding, we studied to what extent Adriamycin could be washed out of the cells. The drug concentration after 1 h rinsing is probably the firmly bound moiety. Its concentration curve is shifted parallel to the total concentration curve. Thus, neither the bound nor the exchangeable compartments will explain the shape of the dose-response curve of Chinese hamster cells to increasing exposure times to Adriamycin *in vitro*.

I thank Dr K. R. Trott, Dr J. Kummermehr and Mrs G. Preuss for helpful discussions during the course of this work. I thank Miss Ingrid Fuhrich for her excellent technical assistance and Montedison Farmaceutica, Freiburg, for kindly supplying the Adriamycin.

### REFERENCES

Barranco, S. C., Gerner, E. W., Burk, K. H. & Humphrey, R. M. (1973) Survival and cell kinetics effects of Adriamycin on mammalian cells. *Cancer Res.*, **33**, 11.

Benjamin, R. S., Wiernik, P. H. & Bachur, N. R. (1974) Adriamycin chemotherapy: Efficacy, safety, and pharmacologic basis of an intermittent single high-dosage schedule. *Cancer Res.*, **33**, 19.

Born, R. (1974) Zellkinetische Untersuchungen an chronisch hypoxischen Fibroblasten des Chinesischen Hamsters. *Dissertation, Biologische Fakultät der Universität München.*

Carter, S. K. (1975) Adriamycin: A review. *J. Natl Cancer Inst.*, **55**, 1265.

Carter, S. K., Di Marco, A., Ghione, M., Krakoff, J. H. & Mathé, G. (1972) *International Symposium on Adriamycin.* Berlin: Springer Verlag.

Creasy, W. A., McIntosh, L. S., Brescia, T. & 4 others (1976) Clinical effects and pharmacokinetics of different dosage schedules of Adriamycin. *Cancer Res.*, **36**, 216.

Di Marco, A. (1975) Adriamycin (NSC—123127) mode and mechanism of action. *Cancer Chemoth. Rep.*, **6**, 91.

Drewinko, B. & Gottlieb, J. A. (1973) Survival kinetics of cultured human lymphoma cells exposed to Adriamycin. *Cancer Res.*, **33**, 1141.

Eichholtz, H. & Trott, K. R. (1980) Effect of methotrexate concentration and exposure time on mammalian cell survival *in vitro*. *Br. J. Cancer*, **41**, 277.

Goldin, A. & Johnson, R. K. (1975) Experimental tumor activity of Adriamycin (NSC—123127). *Cancer Chemoth. Rep.*, **6**, 137.

Pacciarini, M. A., Barbieri, B., Colombo, T., Broggini, M., Garattini, S. & Donelli, M. G. (1978) Distribution and antitumor activity of Adriamycin given in a high-dose and a repeated low-dose schedule to mice. *Cancer Treat. Rep.*, **62**, 791.

Schwartz, H. S. (1973) A fluorometric assay for Daunomycin and Adriamycin in animal tissues. *Biochem. Med.*, **7**, 396.

Skipper, H. E., Schabel, F. M., Jr, Mellett, L. B. and 4 others (1970) Implications of biochemical, cytokinetic, pharmacologic and toxicologic relationships in the design of optimal therapeutic schedules. *Cancer Chemoth. Rep.*, **54**, 431 .

Twentyman, P. R. (1976) Comparative chemosensitivity of exponential—*versus* plateau-phase cells in both *in vitro* and *in vivo* model systems. *Cancer Treat. Rep.*, **60**, 1719.

Wheeler, K. T., Levin, V. A. & Deen, D. F. (1978) Symposium on the principles of radiation research applied to environmental agents. *Radiat. Res.*, **76**, 441.